# Supplementary material for: Mammalian Arginase Inhibitory Activity of Methanolic Extracts and Isolated Compounds from Cyperus Species
Source: Molecules. 2021 Mar 18;26(6):1694. doi: 10.3390/molecules26061694 (PMC8002983; doi:10.3390/molecules26061694)
Supplement: Supplementary file 1 [file molecules-26-01694-s001.pdf]

SUPPORTING INFORMATIONS

Mammalian Arginase Inhibitory Activity and Vasorelaxant Positive Effect on Thoracic Aorta Rings from Rats of Methanolic Extracts from *Cyperus* Species.

Kamel Arraki,[1] Perle Totoson,[1] Alain Decendit,[2] Andy Zedet,[1] Justine Maroilley,[1] Alain Badoc,[2] Céline Demougeot,[1] and Corine Girard[1,*]

[1] PEPITE EA 4267, FHU INCREASE, University of Bourgogne Franche-Comté, 25000 Besançon, France 1

[2] MIB-UR Oenologie, EA 4577, USC 1366 INRA, University of Bordeaux, ISVV, 33882 Villenave d'Ornon, France

* Correspondence: corine.girard@univ-fcomte.fr

N°	Content	Page
1	**Figure S1.** ^{1}H NMR spectrum of compound **1**	1
1	**Figure S2.** ^{13}C NMR spectrum of compound **1**	1
1	**Figure S3.** HSQC spectrum of compound **1**	2
1	**Figure S4.** HMBC spectrum of compound **1**	2
1	**Figure S5.** ^{1}H-^{1}H COSY spectrum of compound **1**	3
1	**Figure S6.** NOESY spectrum of compound **1**	3
2	**Figure S7.** 1H NMR spectrum of compound **2**	4
2	**Figure S8.** ^{13}C NMR spectrum of compound **2**	4
2	**Figure S9.** HSQC spectrum of compound **2**	5
2	**Figure S10.** HMBC spectrum of compound **2**	5
2	**Figure S11.** ^{1}H-^{1}H COSY spectrum of compound **2**	6
2	**Figure S12.** NOESY spectrum of compound **2**	6

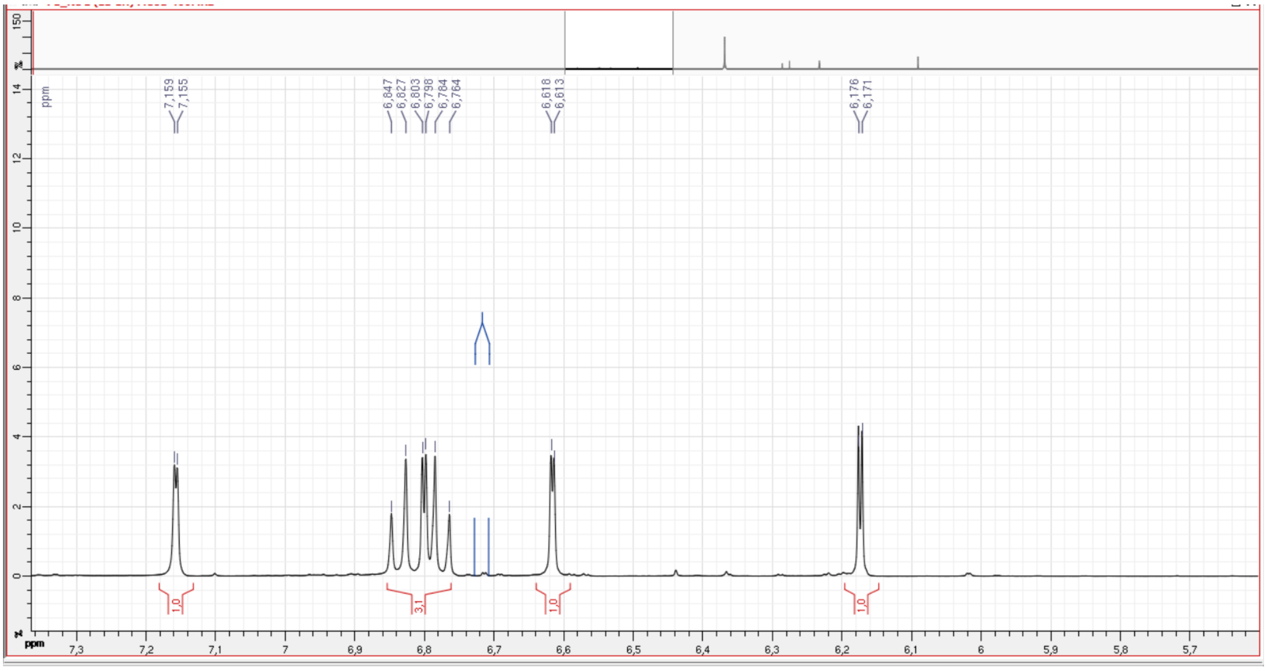

Figure S1. ^{1}H NMR spectrum of compound **1**

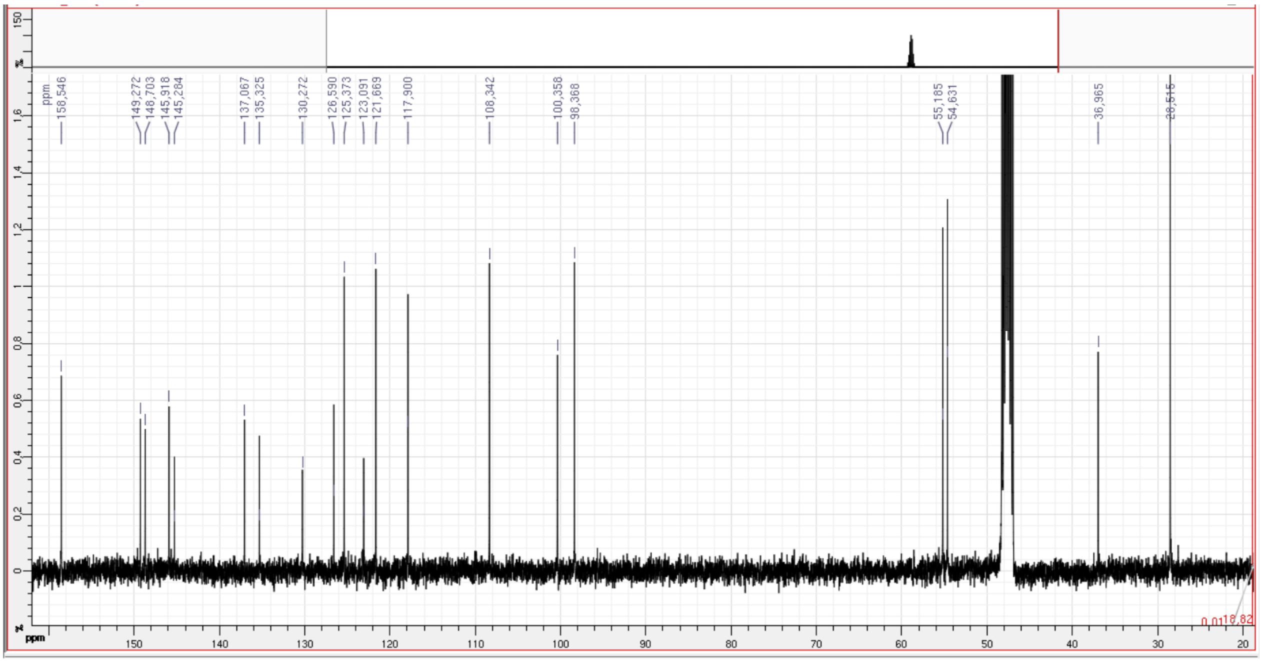

Figure S2. ^{13}C NMR spectrum of compound **1**

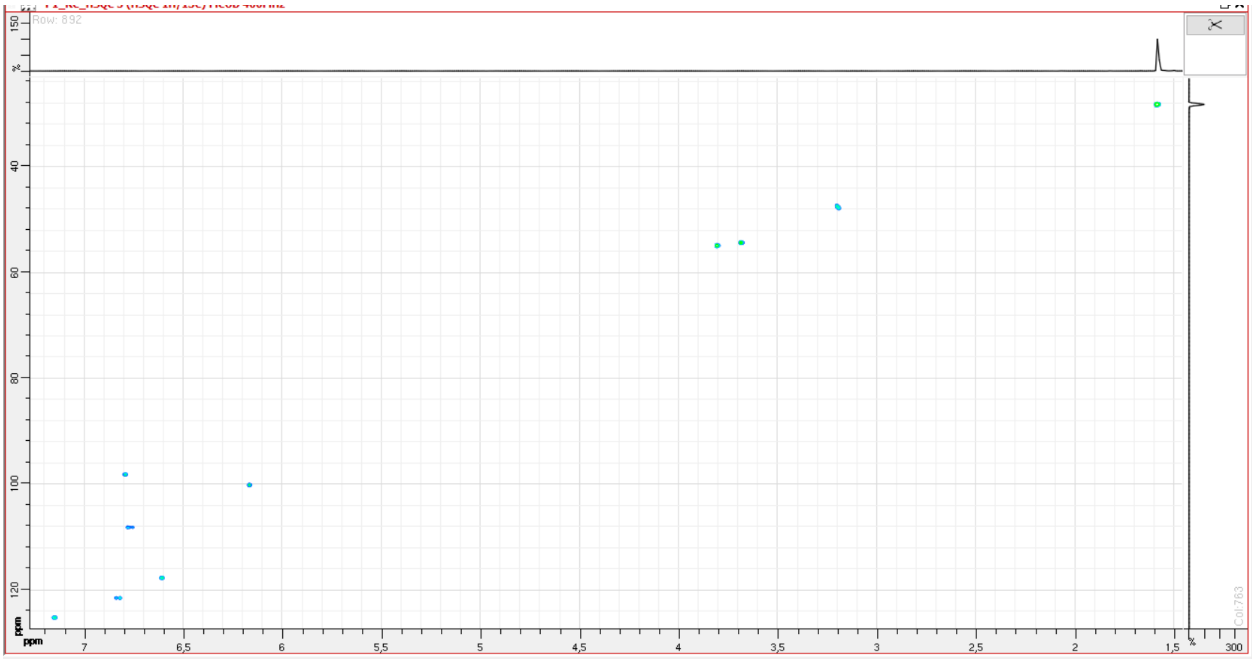

Figure S3. HSQC spectrum of compound **1**

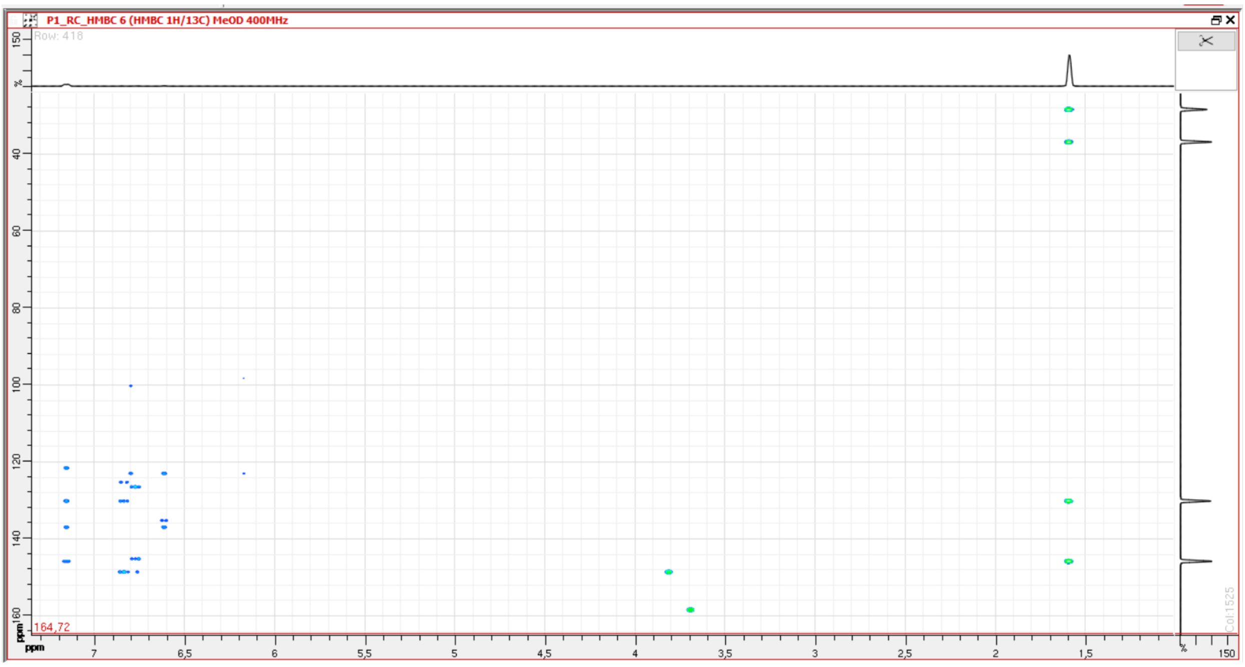

Figure S4. HMBC spectrum of compound **1**

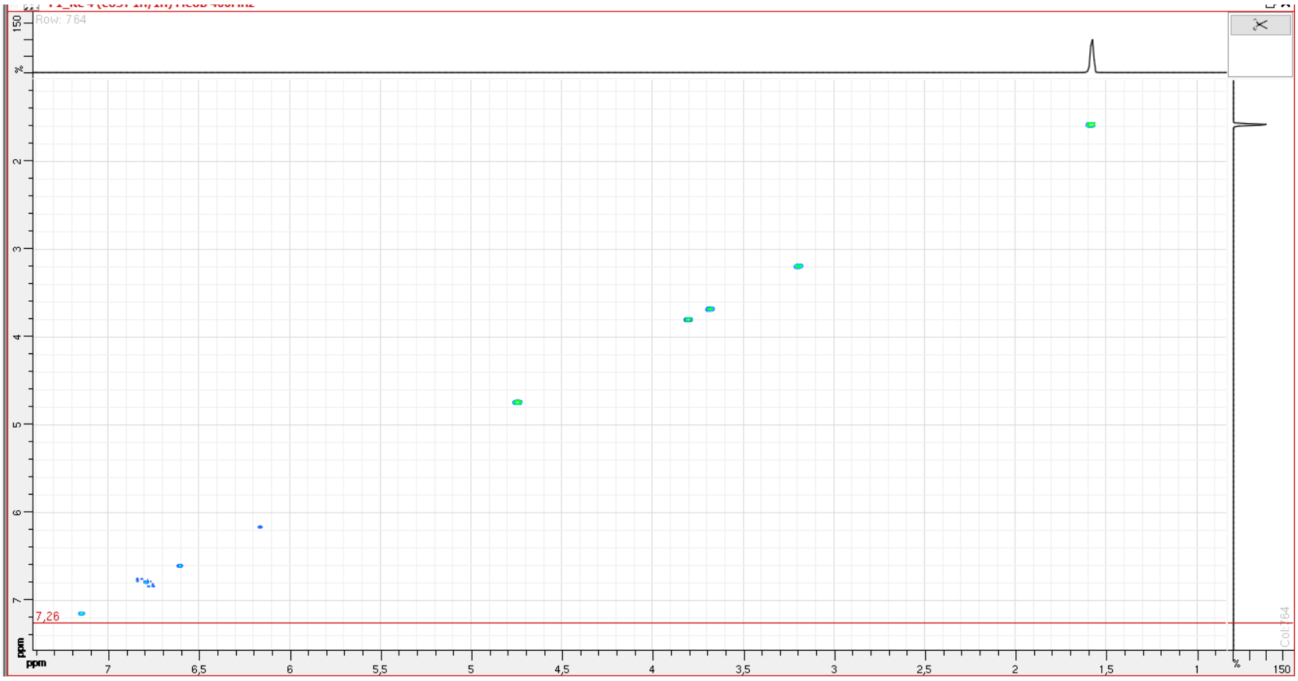

Figure S5. ^{1}H-^{1}H COSY spectrum of compound **1**

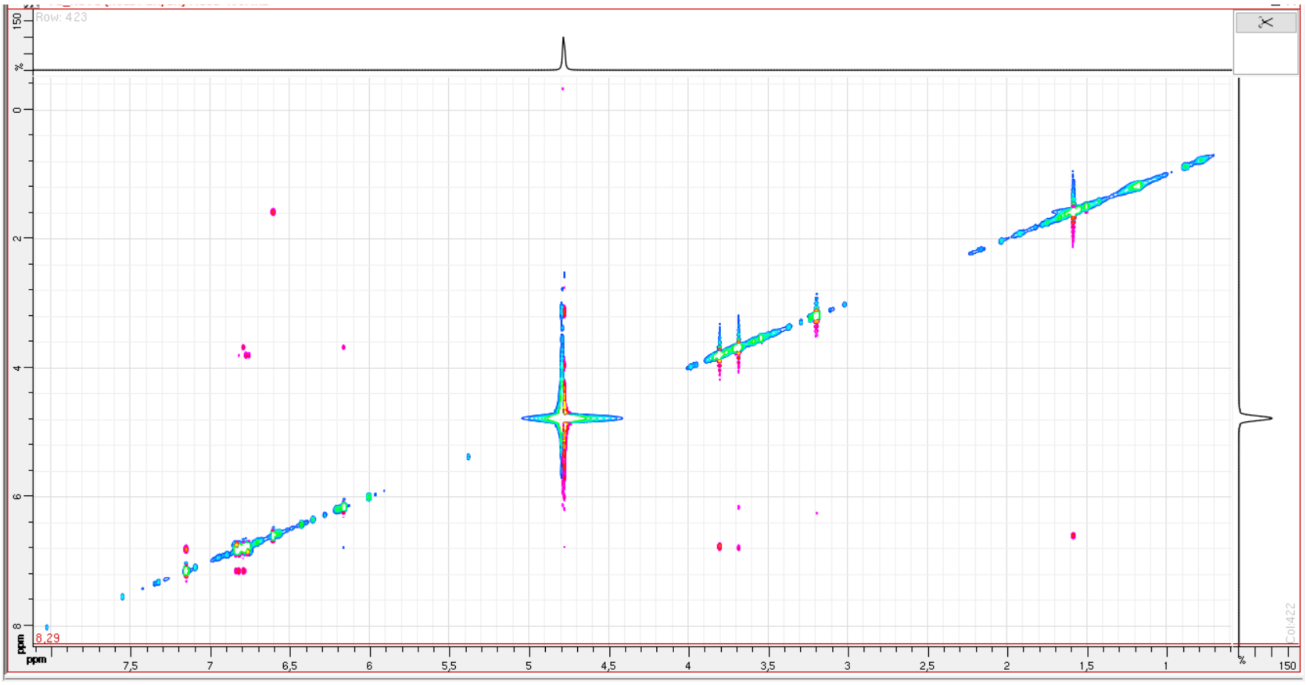

Figure S6. NOESY spectrum of compound **1**

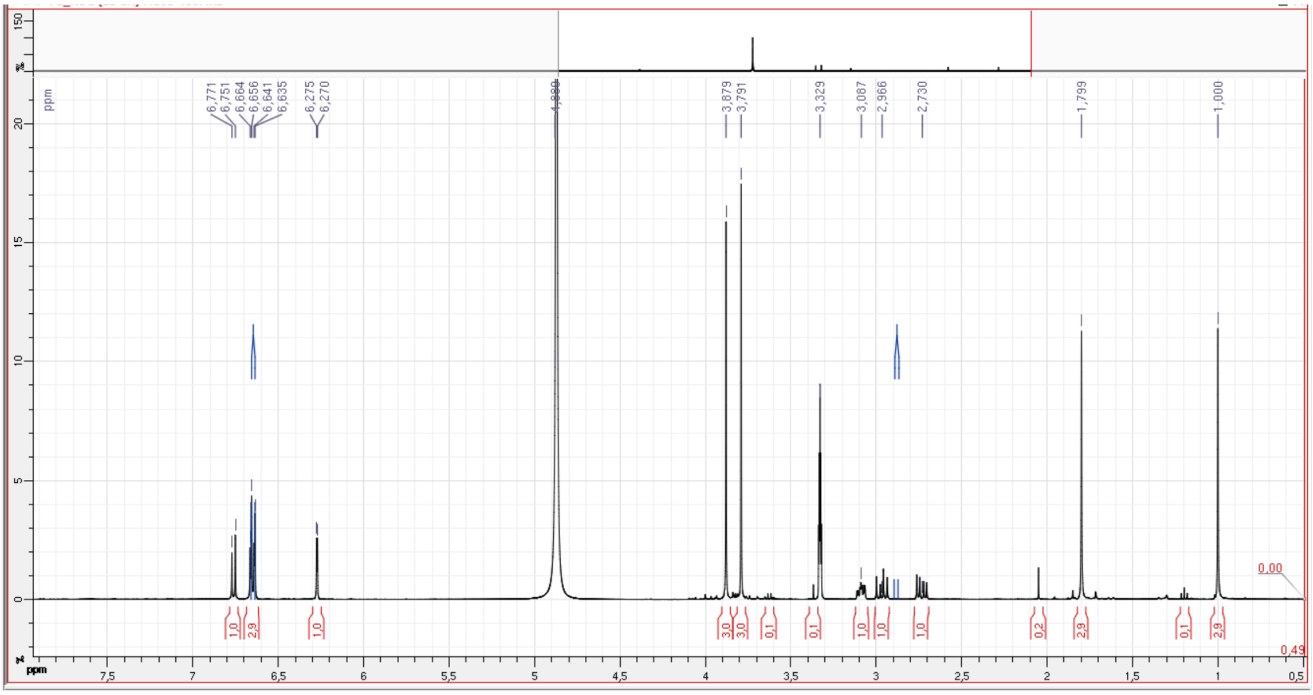

Figure S7. ^{1}H NMR spectrum of compound **2**

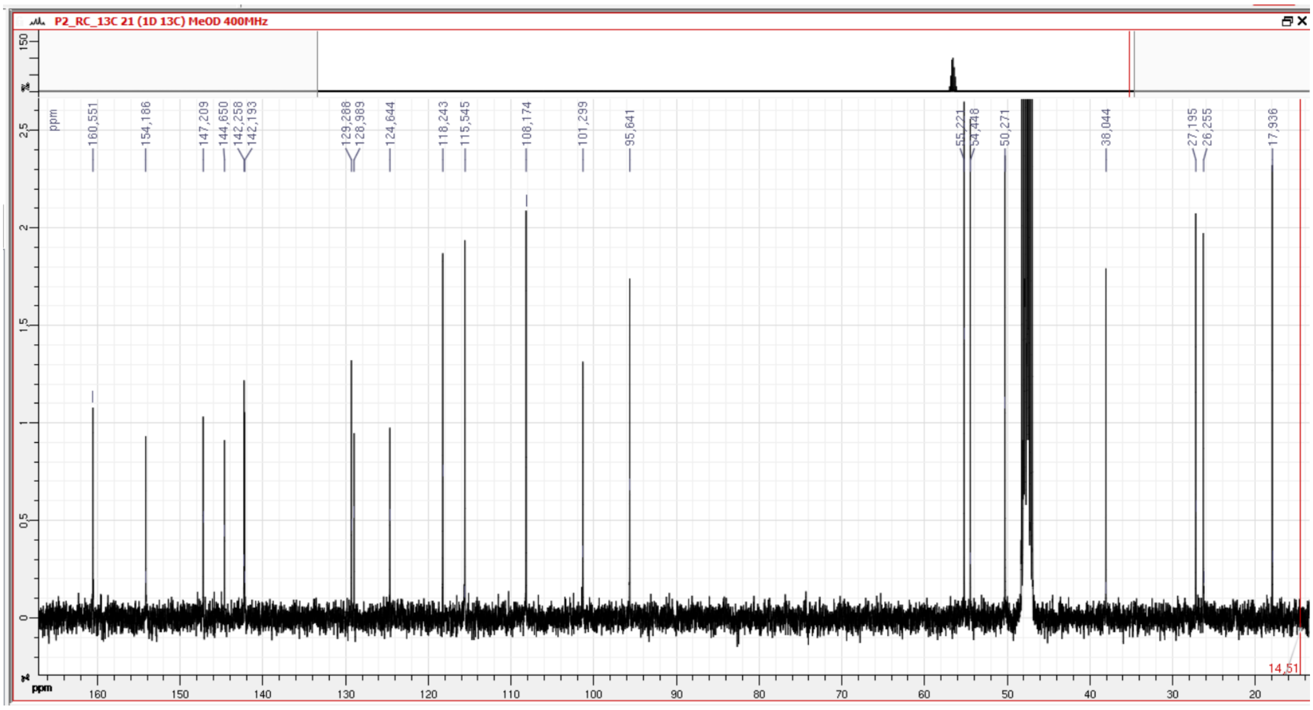

Figure S8. ^{13}C NMR spectrum of compound **2**

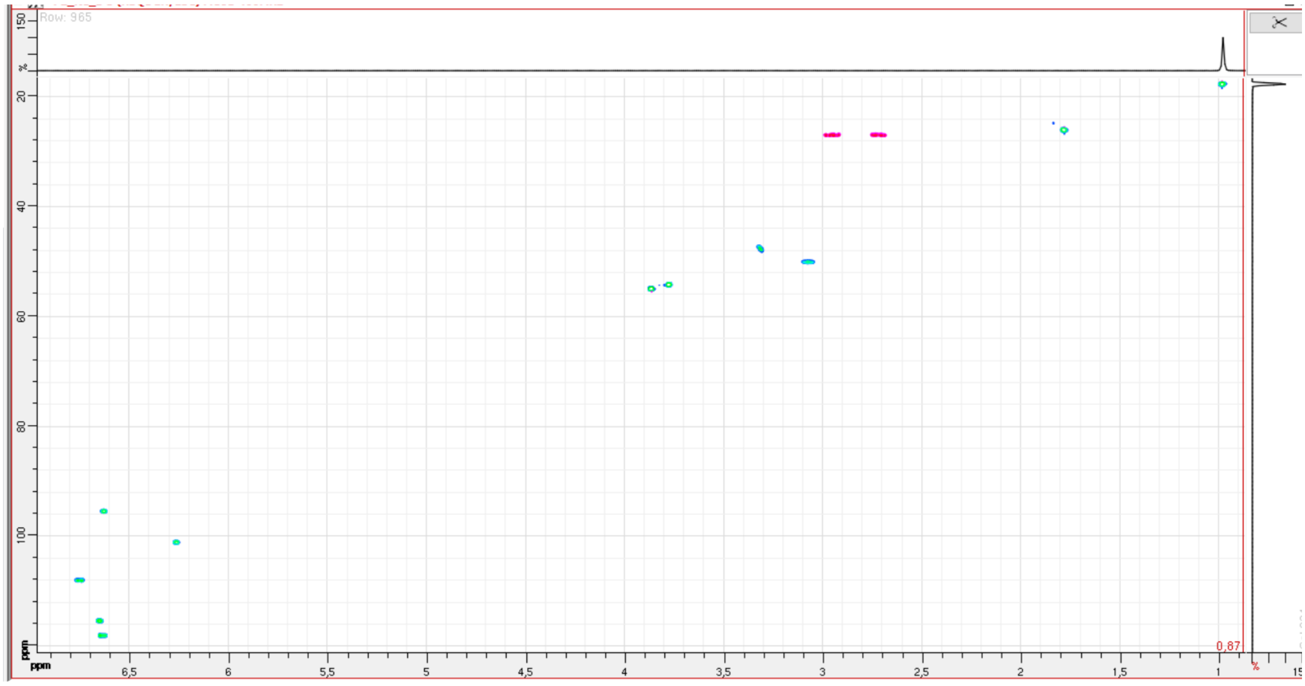

Figure S9. HSQC spectrum of compound **2**

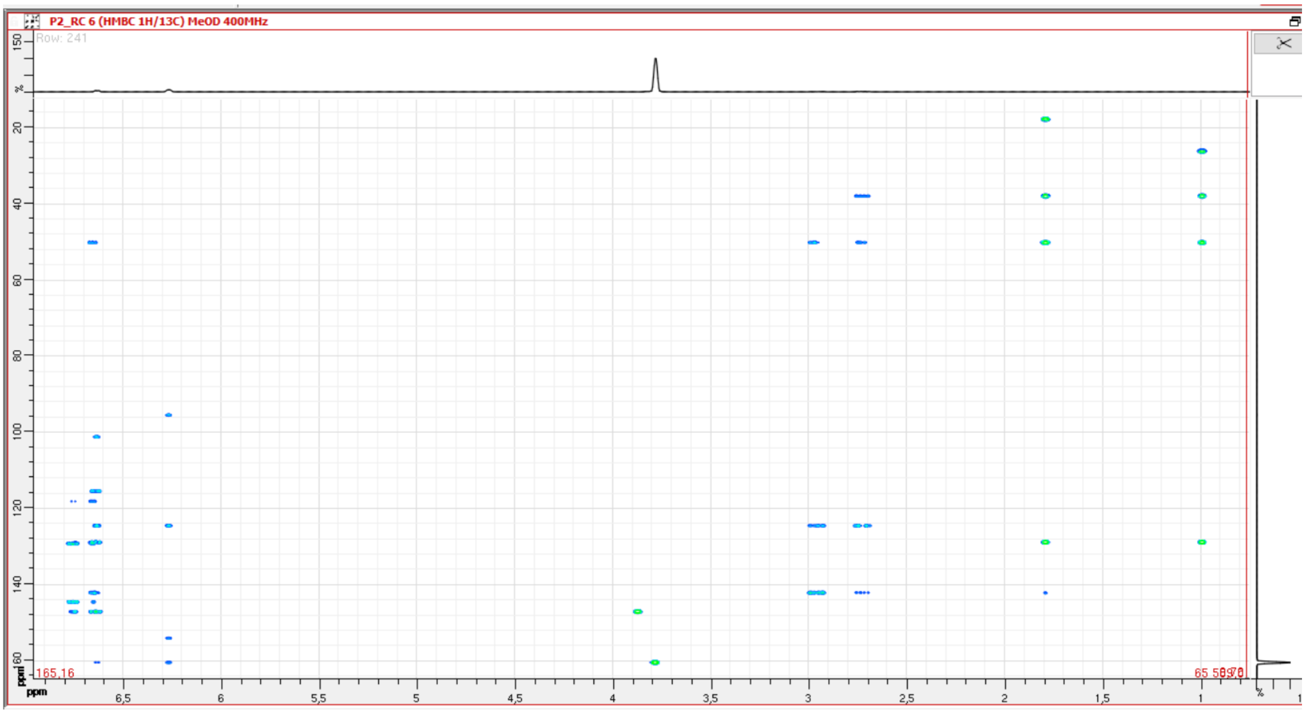

Figure S10. HMBC spectrum of compound **2**

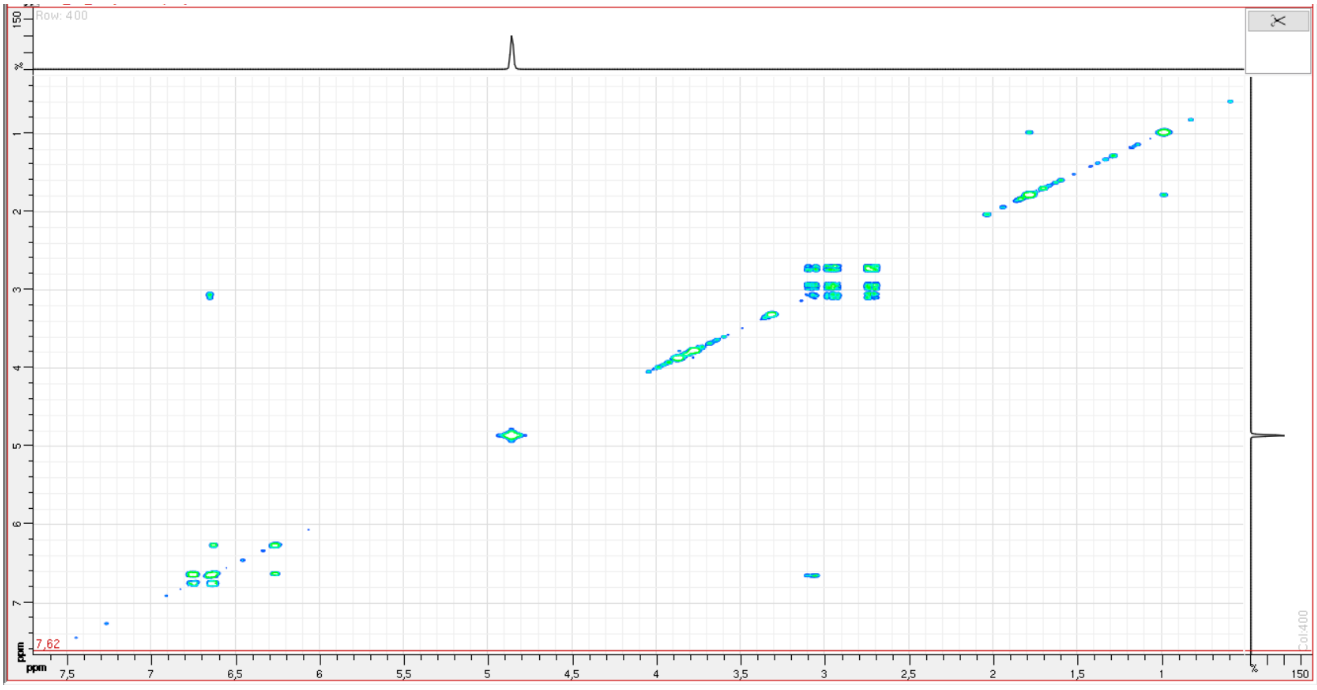

Figure S11. ^{1}H-^{1}H COSY spectrum of compound **2**

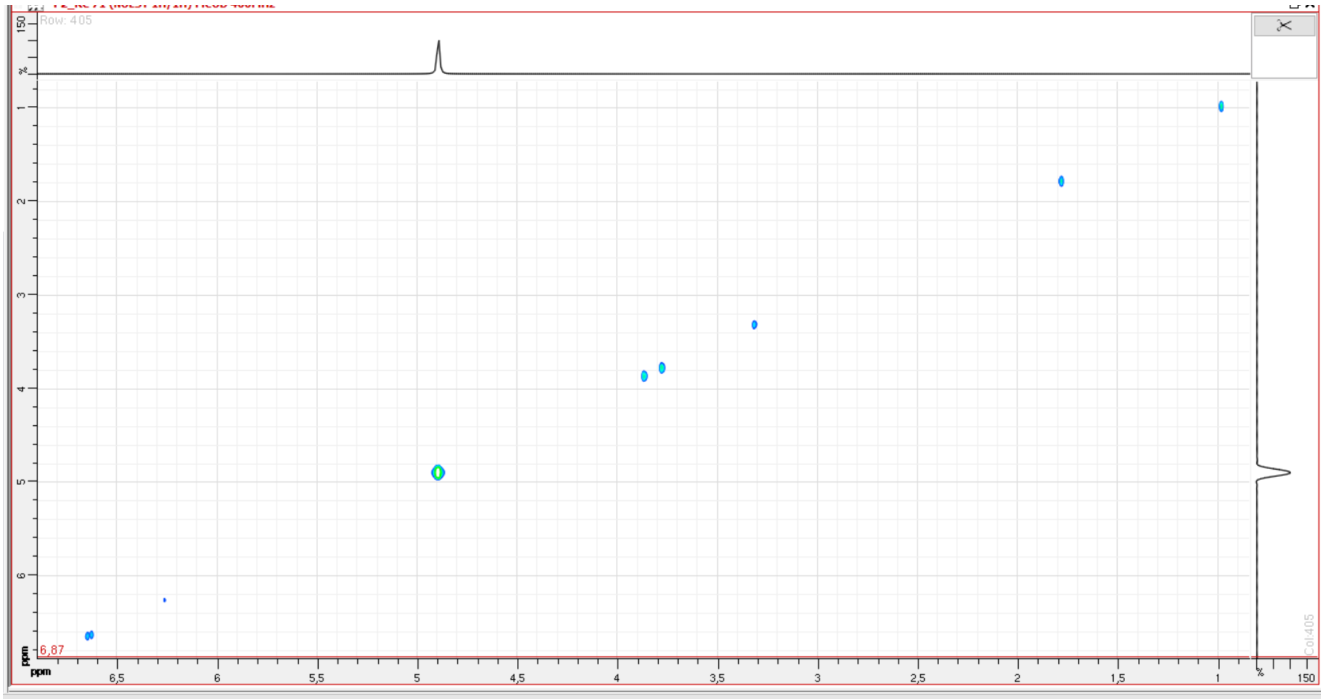

Figure S12. NOESY spectrum of compound **2**
